# Altered Regional Homogeneity in Patients With Diabetic Erectile Dysfunction: A Resting-State fMRI Study

**DOI:** 10.3389/fendo.2022.817523

**Published:** 2022-07-22

**Authors:** Jianhuai Chen, Xinfei Huang, Qinglai Tang, Ziliang Xiang, Yan Xu, Tao Liu, Zhaoxu Yang, Jie Yang, Yun Chen

**Affiliations:** ^1^ Department of Andrology, Jiangsu Province Hospital of Chinese Medicine, Affiliated Hospital of Nanjing University of Chinese Medicine, Nanjing, China; ^2^ Department of Urology, The Affiliated Jiangning Hospital of Nanjing Medical University, Nanjing, China; ^3^ Department of Urology, Jiangsu Provincial People’s Hospital, First Affiliated Hospital of Nanjing Medical University, Nanjing, China; ^4^ Department of Urology, People’s Hospital of Xinjiang Kizilsu Kirgiz Autonomous Prefecture Artux, Xinjiang, China

**Keywords:** type-2 diabetes mellitus, erectile dysfunction, regional homogeneity, resting-state, fMRI

## Abstract

**Introduction:**

Erectile dysfunction (ED) is a common complication of Type-2 Diabetes Mellitus (T2DM) for male patients and it is considered to be associated with different causes including hyperglicemia-induced vascular endothelial cell damages. However, the possible central neural mechanisms shared by these two diseases remain unclear. This study aimed to explore the changes of brain activity and their relationships with the clinical characteristics in patients with diabetic ED.

**Methods:**

The data of resting-state functional magnetic resonance imaging were acquired in 31 T2DM patients with ED (DM-ED) and 31 matched healthy controls (HCs). The whole-brain regional homogeneity (ReHo) values were calculated and compared between groups. In addition, *Pearson* correlation analysis was performed to evaluate the relationships between brain regions with altered ReHo values and clinical characteristics in the patient group.

**Results:**

The DM-ED group exhibited increased ReHo values in the right middle frontal gyrus (orbital part) and decreased ReHo values in the left superior frontal gyrus (dorsolateral), paracentral lobule, precuneus and bilateral supplementary motor area when compared with the HCs group. Moreover, significantly negative correlations were found between ReHo values of the left superior frontal gyrus (dorsolateral) and IIEF-5 scores, as well as the level of HbA1c in the DM-ED group.

**Conclusion:**

The altered spontaneous brain activity in cognitive-related regions revealed by ReHo values might provide new insights into the neurological pathophysiology underlying DM-ED and serve as potential neuroimaging biomarkers for detecting and evaluating ED in diabetes patients.

## Introduction

Type-2 Diabetes Mellitus (T2DM) is a chronic disease, which is characterized by a high morbidity (1, 2). The global diabetes prevalence was estimated to be 10.5% in 2021 (3). More individuals are affected by T2DM and these patients have higher risk of developing cardiovascular diseases and nervous system diseases, especially cognitive impairments (4–8). Diabetes-related cognitive impairments have been found to be associated with the impaired brain function and structure caused by hyperglycemia (9–11). In recent years, increasing attention has been focused on erectile dysfunction (ED) in men with diabetes (12, 13). Individuals with diabetes were found to have increased risk of developing ED compared to individuals without diabetes (14). The prevalence of diabetes in a population of subject diagnosis with ED was 19.5% (15) while the prevalence of ED in patients with diabetes was 52.5% (16). Therefore, ED is considered as the most important sexual dysfunction in patients with diabetes and the prevalence of ED is approximately 3.5-fold higher in patients with diabetes than in those without diabetes (17). Sustained hyperglycemia and long-term fluctuations of glucose concentrations might damage the vascular endothelial cells of the corpus cavernosum in individuals with diabetes, leading to ED (18, 19). Therefore, ED is also considered as a common complication of diabetes and is even considered as an early indicator for diabetes. ED, especially psychological ED (pED) has been found to be closely related to changes of brain structure and function, which included functional abnormalities of regions in the cognitive control and emotional regulation subnetworks and structural abnormalities of regions in the left prefrontal and limbic cortex (20, 21). However, the central pathological mechanisms of ED in diabetes patients have not yet been elucidated.

Human brain is a complex network, which involves in the regulation of multiple functions including perception, emotion and cognition, as well as sexual behavior (22–26). Resting-state functional magnetic resonance imaging (rs-fMRI) is a noninvasive approach for revealing spontaneous brain activity by measuring blood oxygen level dependent (BOLD) signals, which has been broadly applied for exploring the pathogenesis of neuropsychiatric diseases (27, 28). There is no specific task to perform at rest, group differences in the brain activity are thought to reflect intrinsic and unbiased differences in the underlying central neural mechanisms between patients and healthy controls (HCs), rather than group differences in response to the task itself (29). Regional homogeneity (ReHo) is a rs-fMRI measure, which has been widely used to assess the temporal synchrony of blood oxygen level dependent (BOLD) signals within local brain regions (30). The intrinsic brain activity revealed by ReHo is manifested in clusters of voxels rather than in a single voxel, and hence the measure of ReHo may be more accurate (31). In addition, ReHo can inform structure-function relationships for understanding the organization of brain (32).

Patients with T2DM were found to have increased ReHo values in the left superior temporal and angular gyrus (33). Moreover, T2DM patients with mild cognitive impairments showed increased ReHo values in the right inferior frontal gyrus (triangular part), rectus gyrus and decreased ReHo values in the right inferior temporal gyrus, left inferior occipital gyrus, middle occipital gyrus (33). Longitudinal changes of ReHo values were found in patients with T2DM, which mainly distributed in four regions including the left insula, postcentral gyrus and right rolandic operculum, precentral gyrus (34). These findings demonstrated that T2DM might lead to functional impairments in specific brain regions, contributing to cognitive impairments.

The method of rs-fMRI has also been used to explorer the alterations of brain activities in pED (20, 35–37). Patients with pED were found to have decreased amplitude of low-frequency fluctuations (ALFF measuring the spontaneous activities of brain regions) in the left dorsolateral prefrontal cortex, as well as reduced functional connectivity (FC measuring the synchronization of different brain regions) between the left dorsolateral prefrontal cortex and angular gyrus, and the left posterior cingulate cortex and precuneus (37). Altered activities of the right anterior insula and aberrant connection patterns between the right anterior insula and dorsolateral prefrontal cortex, temporoparietal junction were also found in pED patients (35). In addition, pED patients showed decreased functional connectivity density and strength (38). All these results demonstrated that aberrant activities of certain regions in the brain of pED might be related to the clinical features of ED and disrupted psychosocial status.

Based on the findings of previous studies, we hypothesized that diabetic patients with ED might have impaired brain activities in some regions, which might be associated with their clinical characteristics including ED and hyperglycemia. The present study aimed to investigate the alterations of spontaneous brain activity in T2DM patients with ED (DM-ED) using the measure of ReHo with rs-fMRI data.

## Materials and Methods

### Participants

This study was approved by the ethics committee of Jiangsu Province Hospital of Chinese Medicine, Affiliated Hospital of Nanjing University of Chinese Medicine. All participants were informed of the study protocol and signed informed consent before participating in the study.

A total of 31 DM-ED male patients were recruited from the Department of Andrology, Jiangsu Province Hospital of Chinese Medicine, Affiliated Hospital of Nanjing University of Chinese Medicine. In addition, 31 age and education level matched HCs were enrolled at the same time from the local community by advertising. Each participant received a detailed medical history interview, especially history of past illness, and neurological examination for excluding any positive sign. Moreover, the demographic and clinical information were collected from all subjects. The level of fasting plasma glucose (FPG) and hemoglobin A1c (HbA1c) were collected for the diagnosis and evaluation of T2DM while the scores of 5-item International Index of Erectile Function (IIEF-5) were collected for the diagnosis and evaluation of ED (39). The detailed demographic and clinical information of subjects was shown in **Table 1**.

**Table 1:** Demographic and clinical characteristics of DM-ED and HC groups.

Characteristics	DM-ED (n=31)	HCs (n=31)	Statistics	*P*
**Age (years)**	43.48 ± 9.70	41.94 ± 7.73	0.70	0.49^a^
**Gender (M/F)**	31/0	31/0	–	–
**Educational level (years)**	14.45 ± 2.63	14.16 ± 1.75	1.24	0.22^b^
**IIEF-5 (scores)**	15.23 ± 3.15	22.81 ± 0.75	-6.83	<0.01^b^
**HbA1c (%)**	9.52 ± 2.52	5.00 ± 0.48	6.77	<0.01^b^
**FBG (mmol/L)**	9.67 ± 2.76	5.06 ± 0.51	6.77	<0.01^b^

DM-ED, T2DM patients with erectile dysfunction (ED); HCs, healthy controls. M, male; F, female. IIEF-5, 5-item International Index of Erectile Function. HbA1c, hemoglobin A1c; FBG, fasting blood glucose. ^a^ indicated that P values were obtained from two-tailed independent samples t-test; ^b^ indicated that P values were obtained from Mann-Whitney U tests. P<0.05 indicated statistically significant differences.

The inclusion criteria for all participants were as follows: (1) Han Chinese and native Chinese speakers; (2) right-handed; (3) aged 20-60 years; (4) at least 9 years of education. Additional inclusion criteria for patients: (1) had chief complaints of erectile dysfunction and met DSM-V criteria for ED (IIEF-5 scores ≤ 21) (40); (2) diagnosed as T2DM according to the American Diabetes Association (ADA) in 2014 (HbA1c>6.5% or FPG>126mg/dL (7.0mmol/L) or Two-hour PG>200mg/dL (11.1mmol/L) during an OGTT or a random plasma glucose>200mg/dL (11.1mmol/L)) within 2 years (41). Additional inclusion criteria for HCs: (1) IIEF-5 scores>21; (2) FPG<7.0 mmol/L and HbA1c<6.5.

The exclusion criteria for participants were as follows: (1) clinically obvious complications associated with diabetes including cognitive impairments, such as dyschronism and disorientation; (2) any history of severe hypoglycemia; (3) severe neurological and psychiatric diseases or other severe systemic diseases; (4) history of brain injury such as tumor or stroke; (5) alcohol or drug abuse; (6) any contraindications to MRI.

### MRI Data Acquisition

MRI data of all participants were acquired on a 3.0 Tesla Siemens MRI scanner (Version: syngo MR B17, Siemens AG, Erlangen, Germany). All participants were instructed to relax, think of nothing in particular, stay awake with eyes closed, move as little as possible during the scan. Sagittal 3D T1‐weighted images were acquired with the following parameters: repetition time (TR)=1900ms; echo time (TE)=2.48ms; flip angle (FA)=9°; field of view (FOV)=250mm×250mm; matrix size=256×256; slice thickness=1mm; number of slices=176 (42, 43). Rs-MRI BOLD images were acquired with the following parameters: TR/TE =3000/40ms; FA=90°; FOV=240mm×240mm; matrix size=64×64; slice thickness=4mm; number of slices=32; number of volumes=133 (42, 43). All subjects had no obvious structural damages examined by two radiologists based on conventional MRI images.

### MRI Data Preprocessing

MRI Data preprocessing was performed using the software of Data Processing Assistant for Resting-State fMRI (DPARSF) (Advanced Edition; State Key Laboratory of Cognitive Neuroscience and Learning, Beijing Normal University, Beijing, China), a software plug-in within Data Processing & Analysis for Brain Imaging (DPABI), a software plug-in within Data Processing & Analysis for Brain Imaging (DPABI) (44). The steps of MRI data preprocessing were described in our previous studies (42, 43). The first 6 volumes of functional time points were discarded for magnetization stabilization and adaptation of participants. Then, the rest of the volumes were processed as follows: (1) slice timing corrected for acquisition time delay between slices; (2) realignment corrected for head motion between time points (participants with head-motion >2.0 mm or rotation was >2.0° were excluded); (3) T1 images were skull-stripped, co-registered to functional images, segmented into grey matter (GM), white matter (WM), and cerebrospinal fluid (CSF); (4) spatial normalization to the Montreal Neurological Institute (MNI) coordinate space with 3×3×3 mm using the coregistered T1 images by DARTEL; (5) linear detrending; (6) temporal band-pass filtering (0.01-0.08 Hz); (6) regressed out nuisance signals, including Friston-24 head motion parameters, global signal, white matter signal and cerebrospinal fluid (CSF) signal.

### Calculations of ReHo Values

ReHo (30, 45) was calculated using the software of DPARSF. Individual ReHo map of each participant was obtained by calculating Kendall’s coefficient concordance (KCC) of the time series of a given voxel with its 26 neighbor voxels, and was then divided by the global mean KCC value. The measure of KCC was calculated as the correlation between the time series of a given voxel and those of its nearest neighbors in a voxel-wise manner. Finally, standardized map was spatially smoothed with a 6mm×6mm×6mm FWHM Gaussian kernel, which could decrease spatial noise. For subsequent statistical analysis, ReHo map was standardized using Fisher’s r-to-z transformation, which could improve the normality of the correlations.

### Statistical Analysis

The quantitative data were presented as mean ± standard deviation (SD). Data normality was evaluated by the Kolmogorov-Smirnov test. The differences of demographic and clinical variables between groups were compared with two sample *t*‐test or Mann-Whitney U tests using the Statistical Package for the Social Sciences version 23.0 (SPSS Inc, Chicago, IL, United States). *P* <0.05 was considered statistically significant differences.

In addition, two sample *t*‐test (two tailed) was used to compare the differences of ReHo values between groups using the REST Software (version: V1.8; State Key Laboratory of Cognitive Neuroscience and Learning, Beijing Normal University, Beijing, China) (46). The significant difference was set at voxel *P*<0.001 and cluster *P*<0.05 [a minimum cluster size of 6 voxels, corrected by the AlphaSim program (47)].

## Results

### Demographic and Clinical Characteristics Between Groups

No significant differences were found in the age (*P*=0.49) and educational level (*P*=0.22) between groups (**Table 1**). Compared with HCs, DM-ED patients had lower IIEF-5 scores (*P*<0.01) and higher level of HbA1c (*P*<0.01) and FBG (*P*<0.01) (**Table 1**).

### Differences of ReHo Values Between Groups

**Figure 1:**
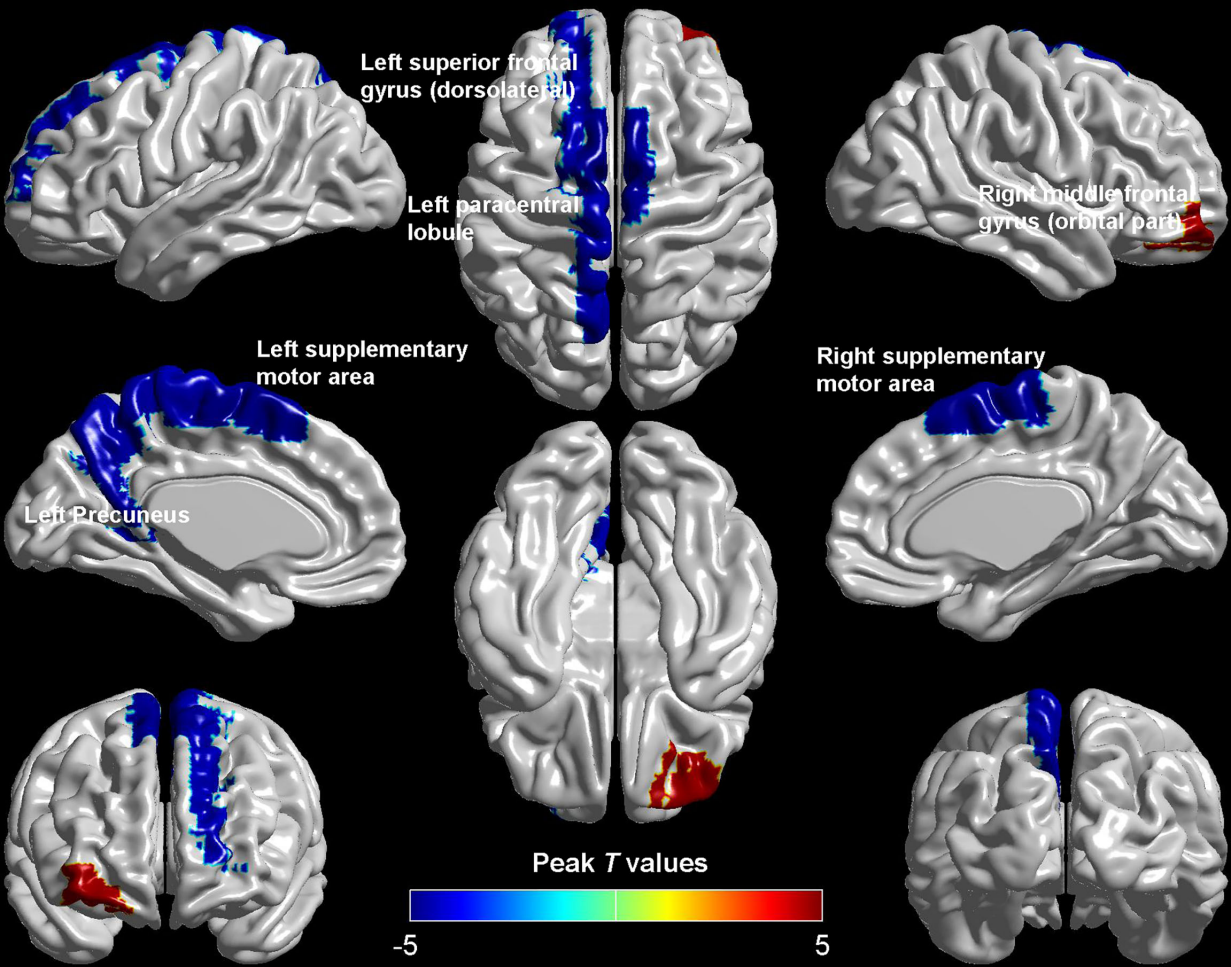
Brain regions showed differences in ReHo values between DM-ED and HC groups.

Compared with HCs, patients with DM-ED exhibited increased ReHo values in the right middle frontal gyrus (orbital part) and decreased ReHo values in the left superior frontal gyrus (dorsolateral), paracentral lobule, precuneus and bilateral supplementary motor area (**Table 2** and **Figure 1**).

**Table 2:** Brain regions showed differences in ReHo values between DM-ED and HC groups.

Brain regions (AAL)	Brodmann area (BA)	Peak MNI coordinates			Clusters	Peak *T* values
		x	y	z		
Right middle frontal gyrus (orbital part)	BA11	30	57	-15	11	4.45
Left superior frontal gyrus (dorsolateral)	BA6	-21	3	72	8	-4.22
Left paracentral lobule	BA6	-9	-24	78	56	-4.29
Left supplementary motor area	BA6	-3	-3	78	51	-4.67
Right supplementary motor area	BA6	9	-12	78	33	-4.56
Left Precuneus^1^	BA7	-12	-54	72	26	-4.42
Left Precuneus^2^	BA7	-9	-75	57	8	-3.78

DM-ED, T2DM patients with erectile dysfunction (ED); HCs, healthy controls. AAL, anatomic automatic labeling; MNI, Montreal Neurological Institute; x, y and z: the coordinates of peak voxel of each cluster in the MNI space.

^1,2^Indicated two activated clusters in the left precuneus.

### Relationships Between Altered ReHo Values and Clinical Characteristics in the DM-ED Group

**Figure 2:**
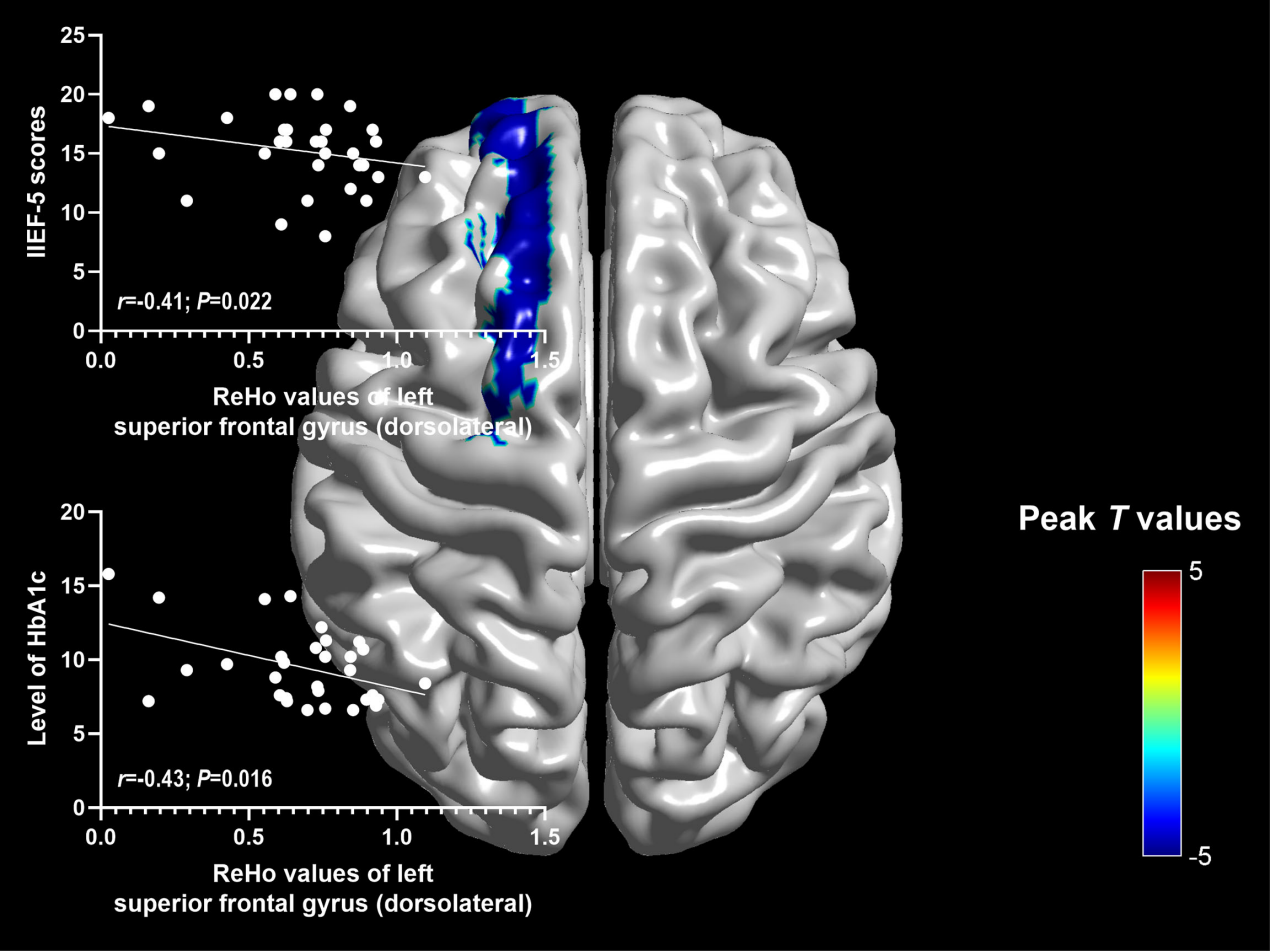
Relationships between altered ReHo values and clinical characteristics in DM-ED group.

Significantly negative correlations were found between ReHo values of the left superior frontal gyrus (dorsolateral) and IIEF-5 scores (r=-0.41; P=0.022), as well as the level of HbA1c (r=-0.43; P=0.016) (**Figure 2**).

## Discussion

To our knowledge, this was the first study to explorer the spontaneous brain activity of DM-ED patients with the measure of ReHo by the method of rs-fMRI. Patients with DM-ED showed altered activity in the cognitive-related regions, including increased ReHo values in the right middle frontal gyrus (orbital part) and decreased ReHo values in the left superior frontal gyrus (dorsolateral), paracentral lobule, precuneus and bilateral supplementary motor area. In addition, ReHo values of the left superior frontal gyrus (dorsolateral) were negatively related to IIEF-5 scores and the level of HbA1c. These findings improved our understanding of the central neural mechanisms of ED in diabetes patients.

ReHo is a measure of neural activity based on BOLD signals and it indirectly reflects local spontaneous neuronal synchronization (30). In 2014, a previous rs-fMRI study used ReHo to investigate global spontaneous activity in patients with T2DM and indicated that T2DM patients had significantly decreased ReHo values in the left postcentral gyrus, bilateral lingual gyrus, left cuneus, right fusiform, right calcarine cortex, bilateral thalamus, bilateral caudate and increased ReHo values in the left medial frontal gyrus and right posterior lobe of cerebellum (48). In addition, these brain regions were related to the impaired cognitive performance of T2DM patients (48). Another study showed that T2DM patients exhibited higher ReHo values in the bilateral anterior cingulate gyrus and lower ReHo values in the right fusiform gyrus, right precentral gyrus and right medial orbit of the superior frontal gyrus (49). Patients with T2DM has been considered to have an increased risk of cognitive impairments (50–54). T2DM patients with cognitive impairment showed decreased ReHo values in the left middle occipital gyrus, inferior occipital gyrus, right inferior temporal gyrus and increased ReHo values in the right inferior frontal gyrus (triangular), rectus gyrus when compared with T2DM patients with no cognitive impairments (33). Moreover, ReHo values of the left cuneus were negatively correlated with HbA1c level while ReHo values of the superior frontal gyrus (medial orbital) and right middle frontal gyrus were negatively correlated with the neurocognitive assessments (33).

T2DM patients had decreased cerebral blood flow in the precuneus (55). Patients with T2DM also showed significantly decreased functional connection in the left superior frontal gyrus, middle frontal gyrus, precuneus and right paracentral lobule when compared with HCs, and the functional connection values of left precuneus were negatively correlated with HbA1c in the T2DM patients (56). Therefore, the alterations of precuneus and frontal regions in this study might be linked to the cognition impairment (decline in memory, attention, executive control, etc) of patients and this relationship would be explored in our further study with data of cognitive assessment, such as the scales of Mini Mental State Exam (MMSE) and Montreal Cognitive Assessment (MoCA). In addition, the abnormality of paracentral lobule was found to be correlated with the disease duration in patients with type 1 diabetes in previous study (57). Patients with T2DM showed a significantly decreased functional connection in the right paracentral lobule (56). T2DM patients also showed reduction in the cortical thickness of the paracentral lobule (58). Moreover, T2DM patients showed lower functional connectivity strength in the right supplementary motor area (59). The supplementary motor area was activated during cognitive task in T2DM patients with mild cognitive impairment (10). These findings indicated that brain changes in these regions might had a great significance in the cognitive impairment of DM patients.

Previous rs-fMRI studies mostly focused on exploring the relationships between T2DM patients with normal or impaired cognition (10, 60). In this study, T2DM patients had clinical changes of erectile function, which suggested that abnormal neural activity of T2DM patients might be also associated with erectile dysfunction. Our previous study showed that the functional brain network of patients with pED exhibited altered functional connectivity in the right superior frontal gyrus (dorsolateral), which located in the cognitive control subnetwork (20). In addition, pED patients were found to have decreased brain activity in the right orbitofrontal cortex, which implied that impaired cognitive processing of sexual stimuli might be associated with ED (36). Altered functional connection patterns of the right dorsolateral prefrontal cortex were found in pED patients, which might also affect the inhibitory control in the sexual context of patients (35). In patients with pED, associations were found between their disrupted psychosocial status and decreased brain activity in the left dorsolateral prefrontal cortex, which suggested that the disrupted psychosocial status mediated the influence of functional connectivity of dorsolateral prefrontal cortex on decreased erectile function (37). In this study, decreased ReHo values were found in the left superior frontal gyrus (dorsolateral) of DM-ED patients. The activity of lateral superior frontal cortex was considered to be associated with erection, which could be enhanced by apomorphine (61, 62). Decreased activity was found in right superior frontal gyrus (dorsolateral) of pED patients in previous study while decreased activity was found in the left superior frontal gyrus (dorsolateral) of DM-ED patients in this study. This finding suggested that the superior frontal gyrus (dorsolateral) might be involved in the pathological mechanisms of ED including psychological factors related ED and diabetes-related ED.

However, there were several limitations in this study. Firstly, the generalizability of findings was limited due to the relatively small sample size. Secondly, we were unable to determine causal relationships between the altered brain activity and clinical characteristics of patients due to the cross-sectional design. Thirdly, the interference of treatment on the results could not be ruled out. Finally, the relationship between testosterone and ED and diabetes had not been analyzed due to the lack of testosterone data. Therefore, further follow-up studies with lager sample size and more clinical data should be performed to explore the central neural pathological mechanisms of DM-ED.

## Conclusion

In conclusion, our findings demonstrated that DM-ED patients had altered spontaneous brain activity in cognitive-related regions. The results suggested that these regions might be involved in the neuropathological mechanisms of DM-ED, which provided a new direction to investigate ED in T2DM patients from the perspective of neuroimaging.

## Data Availability Statement

The original contributions presented in the study are included in the article/supplementary material. Further inquiries can be directed to the corresponding authors.

## Ethics Statement

The studies involving human participants were reviewed and approved by the ethics committee of Jiangsu Province Hospital of Chinese Medicine, Affiliated Hospital of Nanjing University of Chinese Medicine. The patients/participants provided their written informed consent to participate in this study.

## Author Contributions

JC, YC, XH and JY designed the experiments. JC, YC, XH, JY, QT, ZX, YX, TL and ZY contributed to clinical data collection and assessment. JC, XH, JY and QT analyzed the results. JC, XH, JY and QT wrote the manuscript. JC, YC, XH and JY approved the final manuscript.

## Funding Information

The work was supported by the grants of: National Natural Science Foundation of China (No. 81701433; 81871154); Key project of Jiangsu Provincial Health Commission (No. ZDA2020025); Natural Science Foundation of Nanjing University of Chinese Medicine (No. XZR2020003); Special Project of Innovation and Development Fund of Jiangsu Province Hospital of Chinese Medicine (No. Y2021CX24); Jiangsu Province Hospital of Chinese Medicine Project (No. Y21008); General project of Natural Science Foundation of Xinjiang Uygur Autonomous Region (2022D01A23); Key project of scientific research development fund project of Kangda College of Nanjing Medical University in China (No. KD2019KYJJZD020).

## Conflict of Interest

All authors declared that they had no conflict of interest.

## Publisher’s Note

All claims expressed in this article are solely those of the authors and do not necessarily represent those of their affiliated organizations, or those of the publisher, the editors and the reviewers. Any product that may be evaluated in this article, or claim that may be made by its manufacturer, is not guaranteed or endorsed by the publisher.
